# Invasive Bacterial Infections in Subjects with Genetic and Acquired Susceptibility and Impacts on Recommendations for Vaccination: A Narrative Review

**DOI:** 10.3390/microorganisms9030467

**Published:** 2021-02-24

**Authors:** Ala-Eddine Deghmane, Muhamed-Kheir Taha

**Affiliations:** Invasive Bacterial Infections Unit, Institut Pasteur, 28 Rue du Dr. Roux, CEDEX 15, 75724 Paris, France; muhamed-kheir.taha@pasteur.fr

**Keywords:** susceptibility, invasive bacterial infections, complement, genetic factors, *Neisseria meningitidis*, *Streptococcus pneumoniae*, *Haemophilus influenzae*, *Streptococcus agalactiae*, group B streptococci

## Abstract

The WHO recently endorsed an ambitious plan, “Defeating Meningitis by 2030”, that aims to control/eradicate invasive bacterial infection epidemics by 2030. Vaccination is one of the pillars of this road map, with the goal to reduce the number of cases and deaths due to *Neisseria meningitidis*, *Streptococcus pneumoniae*, *Haemophilus influenzae* and *Streptococcus agalactiae*. The risk of developing invasive bacterial infections (IBI) due to these bacterial species includes genetic and acquired factors that favor repeated and/or severe invasive infections. We searched the PubMed database to identify host risk factors that increase the susceptibility to these bacterial species. Here, we describe a number of inherited and acquired risk factors associated with increased susceptibility to invasive bacterial infections. The burden of these factors is expected to increase due to the anticipated decrease in cases in the general population upon the implementation of vaccination strategies. Therefore, detection and exploration of these patients are important as vaccination may differ among subjects with these risk factors and specific strategies for vaccination are required. The aim of this narrative review is to provide information about these factors as well as their impact on vaccination against the four bacterial species. Awareness of risk factors for IBI may facilitate early recognition and treatment of the disease. Preventive measures including vaccination, when available, in individuals with increased risk for IBI may prevent and reduce the number of cases.

## 1. Introduction

Invasive bacterial infections (IBI) usually refer to those infections provoked by *Neisseria meningitidis* ((Nm), meningococcus), *Streptococcus pneumoniae* ((Spn), pneumococcus), *Haemophilus influenzae* (Hi) and *Streptococcus agalactiae* (group B *Streptococcus* (GBS)). The major form of these invasive infections is acute bacterial meningitis. However, other clinical forms are also encountered. The term “bacterial meningitis” is frequently used to refer to all invasive infections due to these agents. In 2020, a road map, “Defeating Meningitis by 2030” was endorsed by WHO. This road map includes an ambitious and broad multidisciplinary plan that includes five pillars to control and eradicate invasive bacterial infection epidemics by 2030: (i) diagnosis and treatment; (ii) prevention and epidemic control; (iii) disease surveillance; (iv) support and aftercare for people affected; and (v) advocacy and information. Actions to achieve the specific goal of prevention and epidemic control include the introduction of vaccines against the four causative agents, achieving equal access to these vaccines and maintaining high coverage of targeted population [1].

Risk factors for developing IBI are linked to bacterial factors (virulence factors). Certain genotypes of these bacterial agents have been reported to be more significantly associated to IBI. The virulence traits are frequently associated with growth in the host, evasion of host immunity, persistence in the host and transmission between hosts [2,3,4,5]. Next, there are factors linked to the host that increase its susceptibility to IBI by enhancing acquisition and/or reducing the clearance of bacterial agents. IBI are often due to underlying anatomical or immune disorders, either of which may be inherited or acquired. Improving surveillance and implementation of vaccines will continue to reduce the incidence of IBI in the general population. However, the burden of these infections among subjects with enhanced susceptibility to IBI will increase proportionally. Another factor that also requires analysis is the severity of invasive bacterial infections. Better knowledge of these two facets (susceptibility and severity) of IBI is therefore warranted. Several aspects of these infections require exploring, for instance, little is known about the genotypes of the involved bacterial isolates and whether they differ from bacterial isolates encountered in the general population. Moreover, response to vaccination and vaccine failure in these subjects are less explored than in the general population. The need for special vaccination schedules also requires analysis. In this narrative review, we aim to summarize the genetic and acquired risk factors that increase the susceptibility to and severity of invasive infections related to the four above-mentioned pathogens and to discuss preventive measures under these conditions.

## 2. Method

We performed a search of PubMed with the objective of summarizing the inherited and acquired host factors associated with susceptibility of patients to invasive meningococcal, pneumococcal, *Haemophilus influenzae* and group B streptococci disease. The following Mesh terms were used: ((*Neisseria meningitidis*) OR ((*Streptococcus pneumoniae*) OR (*Haemophilus influenzae*) OR (*Streptococcus agalactiae*) OR (group B streptococc*)) AND (((invasive) AND ((disease*) OR (infection*))) OR (bacterial meningitis) OR (meningitis) AND ((genetic) OR (acquired) OR (immunocompromised)* or (deficien*) OR (immunodeficient*) OR (susceptibility) OR (predispose*) OR (recurrent infection*)). A built-in PubMed filter was used to limit the search to papers published in English or French up until 31 October 2020. Both authors independently screened titles and abstracts. Studies lacking outcomes of interest were considered not relevant to the aim of our review and were excluded. Relevant publications matching the criteria applied to the search results were identified, and the full text of each was reviewed by both authors separately.

## 3. Susceptibility to Invasive Meningococcal Infections

Nm is a human-restricted, Gram-negative encapsulated bacterium that is usually encountered as a member of the nasopharyngeal microbiota, which acts as a carriage. However, a few genotypes (hyper-invasive clonal complexes) are associated with invasiveness of the bloodstream and are responsible for most of the cases of invasive meningococcal disease (IMD). Carriage and hyper-invasive isolates differ genetically and phenotypically. Unlike invasive isolates, carriage isolates are more frequently non-capsulated and do not belong to hyperinvasive genotypes [6]. The incidence of IMD varies according to age, with three peaks: in infants < 1 year of age, in adolescents and young adults and in the elderly. This incidence also varies geographically and the epidemiology of IMD is continuously changing [7,8].

The meningococcal capsule is a polysaccharide, and when present, it determines the serogroup. Twelve serogroups have been described with serogroups A, B, C, W, Y and X being responsible for virtually all cases of IMD [8]. Capsular polysaccharide-based vaccines are available against Nm of serogroups A, C, W and Y, while subcapsular protein-based vaccines are available against Nm of serogroup B. Recommendations exist to use these vaccines in subjects with increased susceptibility to IMD. However, rational support for these recommendations may require clarification.

### 3.1. Genetic and Acquired Susceptibilitiesy to IMD

The ability of Nm to invade, to survive and to spread in the bloodstream is linked to its pathogenesis, which is correlated to the complement-dependent clearance of meningococci. Factors that lead to the absence of bactericidal activity in complement-dependent serum increase the susceptibility to IMD [9,10]. These factors can be inherited and/or acquired.

#### 3.1.1. Inherited Factors of Susceptibility to IMD 

The three pathways of the complement system (the classical, the lectin and the alternative pathways) are major actors in the innate immune response. Activation of complement is tightly controlled with several regulators. Complement is activated through the early complement components of these three pathways to first form C3 convertases, then, they converge to form the C5 convertase, and subsequently, the membrane attack complex (MAC) through the activation of the late complement components (LCC) (C5 to C9). The MAC ultimately leads to the lysis of the targeted cell. Moreover, complement activation leads to the opsonization of the bacterial surface [11]. These two events (lysis and opsonophagocytosis) are directly responsible for efficient bacterial clearance [12]. For Nm, bactericidal activity (in the absence of blood inflammatory cells) is able to lyse bacteria through the insertion of the MAC at the bacterial surface [9,13]. Deficiencies in these late components of the complement system lead, therefore, to enhanced susceptibility to IMD, which can result in repeated IMD [13,14,15]. This is particularly the case in subjects with late components of complement deficiencies (LCCD), deficits of properdin deficiency or deficits of factor D deficiency [15,16]. Polymorphism of Factor H (a negative regulator of the complement) is also associated with an increased risk of IMD while deficiencies in the early components (such as C1) were not reported to be specifically associated with increased susceptibility to IMD [17,18]. The incidence of IMD among LCCD patients, in regard to number and proportion, will increase due to the decreasing incidence of IMD in immune-competent subjects upon implementation of vaccination strategies. The incidence of IMD is 1000 to 10,000 times higher among LCCD patients than among the general population [15]. The frequency of hereditary complement deficiencies varies according to their type, age, sex and geographical/ethnic distribution [15]. Terminal complement pathway, properdin and factor D deficiencies seem to lead specifically to an increased susceptibility to IMD. LCCD are the most frequent but seem to be associated with a low fatality rate (1%), and are usually detected in adolescents and young adults [15,19]. About 45% of these patients developed more than one IMD episode with a median interval of 6 years between episodes of IMD [19]. Meningococcal isolates from IMD in patients with LCCD are often of serogroup Y, non-groupeable isolates or serogroups/genotypes that are rare in typical cases of IMD. Moreover, IMD disease among LCCD patients seems to be less severe with lower mortality than IMD in the general population [15,19,20]. The median age for the detection of LCCD is 17 years and it is frequently suspected due to repeated IMD episodes, while the detection of properdin deficiencies occurs earlier [15]. Moreover, fulminant and fatal IMD in patients with properdin deficiencies has been frequently reported [21,22,23,24]. However, properdin deficiencies are not all complete and there are three types: total deficiency (type I), partial deficiency (type II), and deficiency due to a dysfunctional molecule (type III). 

#### 3.1.2. Acquired Factors of Susceptibility to IMD 

The complement system has two facets and it plays the role of the two characters in the Dr Jekyll and Mister Hyde story. Indeed, complement is a major and beneficial actor in immune response and host defense, however, its over-activation may lead to systemic effects such as systemic lupus erythematosus (SLE, a systemic autoimmune disorder in which multiple autoantibodies against cell nuclear constituents form immune complexes that effectively activate the classical complement pathway and cause tissue damage) [25], paroxysmal nocturnal hemoglobinuria (PNH, an X-linked hematological disorder that results from somatic loss-of-function mutations impairing membrane expression of two complement inhibitors, CD55 and CD59, on red blood cells, resulting in erythrocytes-complement mediated lysis) [26], age-related macular degeneration (AMD, characterized by the progressive destruction of neurosensory retina in the macular area, and which contributes to vision loss) [27] and atypical hemolytic uremic syndrome (aHUS, a disorder related to mutations in complement regulators (such as the factor H), and that result in a renal disease that encompasses the triad of microangiopathic hemolytic anemia, thrombocytopenia, and acute renal failure) [28]. Several of these systemic diseases may benefit from anti-complement drugs, and in particular, monoclonal antibodies (Mabs) that inhibit the late complement components. This inhibition of the complement can therefore increase susceptibility to IMD. Mabs that inhibit the C5 (Eculizumab and Ravulizumab) have reached the market and are used to treat aHUS and PNH. Other drugs are under development, targeting other components such as C3, factor B and factor D [20,29]. Treating COVID-19 with compstatin-based complement C3 inhibitor (AMY-101) has also been reported [30]. The use of anti-complement drugs in the management of various pathologies is growing [31], including the treatment of COVID-19 to control the inflammatory response [32]. IMD frequency in these patients should therefore be kept under tight surveillance.

Other acquired susceptibilities to IMD are encountered in cases of anatomic or functional asplenia. The spleen plays a central role in mounting innate and adaptive immune responses against encapsulated pathogens such as Nm. Asplenia/hyposplenia (including sickle-cell disease) were reported as a recognized risk factor of IMD in a large case-control study (odds ratio, 6.7; 95% confidence interval (CI), 3.0–14.7). Patients with hematopoietic stem cell transplantation (hSCT) are also at high risk for IMD as well as HIV patients [33,34]. hSCT is a procedure in which the immune system is transferred from the donor to the recipient. This transfer is at best incomplete and vaccine protection from the donor is usually lost. This loss is observed in particular, when the patient suffers from a graft-versus-host disease (GVHD) that requires the administration of immunosuppressive treatments [33].

hSCT transplant recipients are at risk of IMD due to total body irradiation, which induces a hyposplenism, and especially the progressive loss of specific antibodies, which has been documented in the literature for meningococci [35]. Solid organ transplant recipients may also be at risk for IMD due to immunosuppressive treatment [36].

### 3.2. Host Factors of Severity of IMD

The severity of IMD is frequently linked to hyperinvasive clonal complexes, and particularly, the clonal complex 11 [37]. However, several host factors are reported to be associated with severity and/or bad evolution of the disease. The deficiency of either protein C or its cofactor, protein S (anticoagulant proteins) has been reported as being associated with an increased risk of severe meningococcal sepsis [38]. Moreover, high levels of the plasminogen activator inhibitor-1 (PAI-1) have been associated with poor outcome of IMD with high sequelae and mortality rates [39]. The exacerbated inflammatory response may lead to complications such as pachymeningitis, which can be linked to promoter variants in genes involved in the inflammatory response (IL6, PAI-1 and macrophage migration inhibitory factor, MIF) [40]. 

### 3.3. Impact on Anti-Meningococcal Vaccination Strategies

Exploring the complement is highly recommended in patients who develop recurrent/chronic forms and/or mild infections provoked by unusual serogroups/genotypes of Nm. This exploration should include assays for C3, C4, CH50 and AP50 in order to detect deficiencies in early and late components and alternative pathways. When detected in a patient, the investigation should be extended to the siblings. LCCD are inherited in an autosomal recessive manner while properdin deficiencies are usually inherited as an X-linked disorder. 

These patients (with acquired or hereditary complement deficiencies) are increasing due to increasing detection and new indications for anti-complement drugs such as Mabs. These drugs are being investigated in the treatment of COVID-19 [41]. Moreover, the number of patients with spleen disorders is substantial, for example, 6000 to 9000 patients are splenectomized each year in France [42].

These patients with increased susceptibility to IMD require particular management strategies including:

- Large-spectrum vaccination against meningococci using conjugate vaccines against serogroups ACWY (with a booster dose every 5 years) and protein-based vaccines targeting serogroup B isolates.
- Exploration of the siblings in case of genetic deficiency (the same management should be proposed for each case detected).
- Reinforcing protection around the patient by vaccination of household contacts (cocooning or barrier) strategy.
- Prophylactic antibiotic treatment is also required using oral penicillin V. For example, penicillin V is recommended in several countries in addition to vaccination for patients receiving anti-C5 treatment.
- Teaching patients to seek immediate medical help if they feel unwell (fever).

The immunogenicity of meningococcal vaccines in these patients requires more exploration in order to adapt vaccination schemes. For example, in a study on adult asplenic patients, they were able to achieve protective bactericidal titers after vaccination against serogroup C meningococci. However, they showed a significantly lower geometric mean titer (GMT) (157.8; 95% CI, 94.5 to 263.3) of bactericidal antibody in serum (SBA) than an age-matched control group (1448.2; 95% CI, 751.1 to 2792.0). The primary vaccination schemes may require several doses in these patients in addition to repeated boosters [43]. Immunogenicity after one dose of tetravalent conjugated ACWY vaccine was also poor in recipients of allogeneic hematopoietic stem cell transplantation [44]. The administration of two primary doses of polysaccharide conjugated anti-meningococcal vaccines is therefore recommended in several countries for patients with asplenia, HIV, or complement disorders [31,45]. No immunogenicity data on vaccines against meningococcal B are available among these subjects.

## 4. Susceptibility to Invasive *H. influenzae* Infections

Like Nm, *H. influenzae* is also a Gram-negative human-restricted encapsulated bacterium that is a member of the nasopharyngeal microbiota. Hi is highly polymorphic with six different capsular types (serotypes a to f) as well as non-capsulated isolates (nontypeable isolates, HiNT). The incidence of Hib infection has been drastically reduced since the introduction of a vaccination against this serotype. Invasive disease due to other serotypes as well as non-typeable isolates persists and no vaccine is available against these non-Hib isolates.

### 4.1. Genetic and Acquired Susceptibilities to Invasive Haemophilus influenzae Disease

As for Nm, disorders that affect the immune defense mechanisms and mainly the complement system are expected to increase susceptibility to invasive *H. influenzae*. The frequency of Hi infection in patients with early component deficiencies (C1, C2, C4) seems to be similar to that of meningococcal infections. However, this frequency is lower in infections in patients with C3 deficiencies and LCCD, suggesting that functions other than the lytic functions of the MAC are involved in the defense against invasive Hi infections. However, Hi invasive infections are still higher among patients with complement deficiencies (including factors P or D) than in the general population [15]. 

Disorders that influence the efficiency of IgG2 binding, the main isotype produced in response to encapsulated bacteria may also increase susceptibility to Hi infections. For example, the His131Arg allele encoding Fcgamma RIIa receptor (rs1801274) binds IgG2 poorly, and therefore, increases the risk of Hi infections [46]. Patients with a single nucleotide polymorphism (SNP) in the *TIRAP* gene (Toll-interleukin 1 receptor domain containing adaptor protein, an adapter molecule associated with Toll-like receptor) (rs1893352) was reported to be strongly associated with non-meningitis cases of Hib in vaccinated children. Another SNP (rs1554286, a promoter SNP in the interleukin-10 encoding gene) was associated with epiglottitis [47]. Patients with asplenia, hSCT, HIV are also at high risk for invasive Hi disease [48].

### 4.2. Impact on Anti-Hi Vaccination Strategies

There is an unmet medical need in the field of vaccination against *H. influenzae* among patients at high risk due to the absence of vaccines against non-Hib isolates, and particularly, non-typeable Hi (NTHi) isolates. Unlike Nm, only vaccines against serotype B are available. New vaccines, immunogenicity knowledge and vaccination strategies are therefore needed. Non-Hib invasive infections can be more prevalent in patients at risk for Hi invasive infections, underlying the need for vaccines against other serotypes and non-typeable isolates of Hi. Moreover, studies on the immunogenicity of Hib vaccine in these patients are lacking; however, the implications of genetic traits on vaccine efficacy have been suggested [49].

## 5. Susceptibility to Invasive Pneumococcal Infections

The Gram-positive bacterium *Streptococcus pneumoniae* is an endemic global pathogen that causes a wide range of non-invasive and potentially life-threatening invasive diseases in children and adults. Invasive pneumococcal disease (IPD) implies invasion of pneumococcus into a normally sterile site, leading to several forms of IPD such as bacteremia, empyema, meningitis, endocarditis, and osteomyelitis [50,51]. The incidence of IPD, which ranges from 11 to 27 per 100,000 in Europe, is highest in younger children and the elderly [52,53,54]. Mortality rates for IPD vary from 12% to 22% in adults in developed countries and are substantially higher in low-income countries. Neurological sequelae, including hearing loss, focal neurological deficits, and cognitive impairment occur in 30–52% of surviving patients [55,56,57,58]. Susceptibility to IPD relates to both the virulence of the pathogen and to host factors. The most relevant host factors responsible for the increased risk of IPD are related to defects involving the immune system [59]. 

### 5.1. Genetic and Acquired Susceptibilities to IPD

Several inherited and acquired host factors have been shown to confer predisposition to IPD. In particular, primary immunodeficiency states, dysfunction or absence of the spleen and human immunodeficiency virus (HIV) infection, confer a high degree of susceptibility to IPD [60]. Recently, increasing evidence supports a central role of the NF-κB pathway in susceptibility to severe IPD [61]. 

#### 5.1.1. Inherited Factors of Susceptibility to IPD

##### Congenital Deficiencies in Immunoglobulins

In contrast to *N. meningitidis* and *H. influenzae* (Gram negative bacteria), the thick cell wall of *S. pneumoniae* (Gram positive) renders it resistant to lysis by insertion of the complement MAC. Furthermore, the presence of a polysaccharide capsule (that can have a thickness of 175 nm in some serotypes) makes them even harder targets for complement-mediated lysis. Antibody-initiated complement-dependent opsonization (opsonophagocytosis), which activates the classic complement pathway, is thought to be the major immune mechanism of pneumococcal killing. Opsonization, refers to the coating of bacteria with antibodies and complement ligands, mainly C3b and iC3b, to facilitate their elimination through phagocytosis by cells bearing complement receptors. Therefore, the production of specific polysaccharide antibodies (IgA, IgM and IgG) and complement activation are the cornerstones to trigger complement-mediated opsonophagocytosis of pneumococci and proper T-B lymphocyte cooperation for an efficient antibody response. Specific antibody deficiencies to *S. pneumoniae* contribute to the increased rates of invasive infection [62]. Although specific rates are not available, patients with agammaglobulinemia (absence of B cell immunoglobulins due to a defect in maturation of B cells) or hypogammaglobulinemia (characterized by reduced serum levels of immunoglobulins and a diminished vaccinal response) are susceptible to invasive *S. pneumoniae* infection [63,64,65]. Specifically, as IgG antibody responses to bacterial capsular polysaccharide antigens are mostly restricted to IgG2, patients with IgG2 deficiency are more susceptible to infections with *S. pneumoniae,* presumably because of the proposed unique ability of IgG2 to support neutrophil phagocytosis of pneumococci in the absence of complement [66,67]. Moreover, hyper-IgM syndromes (HIGM) are a group of hereditary immune system pathologies, characterized by ineffective immunoglobulin class switching, resulting from interrupted B cell co-stimulation. Patients with hyper-IgM have ineffective production of specific IgG and are susceptible to IPD and sepsis [68].

##### Congenital Deficiencies in Complement

Only a few clinically defined groups of patients experiencing pneumococcal disease have been systematically examined for the frequency of complement deficiencies [69]. In particular, it has been shown that certain complement deficiencies predispose patients to pneumococcal infections with, in decreasing order of frequency, the C3, the C2 and the C4 defects [63]. Sporadic pneumococcal infections have been diagnosed in patients with C1 and alternative pathway defects (properdin, factor D or factor I deficiencies) [70]. Findings on the role and the link between Mannose-binding lectin (MBL) deficiency and increased susceptibility to pneumococcal infections are conflicting [71,72,73]. Nevertheless, Eisen et al. analyzed the association between MBL deficiency and the outcome of IPD using data pooled from five studies with adults and one study with children and concluded that the risk of death was increased among MBL-deficient patients with *S. pneumoniae* infection (odds ratio, 5.62; 95% confidence interval, 1.27–24.92) after adjustment for bacteremia, comorbidities and age [74]. MBL deficiency may therefore be considered as a factor of severity instead of a risk factor for developing IPD. 

##### Toll-Like Receptor Signaling Deficiencies

TLR signaling is critically important in the first unspecific meeting between host and microbe. Specific defects of molecules in the TLR signaling pathway including interleukin-1-receptor associated kinase-4 deficiency (IRAK-4), myeloid differentiation factor 88 (MYD88) and nuclear factor-κB essential modulator deficiency (NEMO) [63,75,76,77,78] have recently been defined. IRAK-4, a serine threonine kinase, is essential for signal transduction downstream in TLR canonical pathways. IRAK-4 deficiencies are inherited in an autosomal recessive manner [79,80]. Selective susceptibility to *S. pneumoniae* infections is high and many experience recurrent IPD in early childhood. High mortality (40%) is reported before the age of 8 years; however, among survivors, clinical phenotype of patients with IRAK-4 and MyD88 deficiencies tend to improve with age [79]. 

NF-κB essential modulator (NEMO), encoded by the X-linked *IKBKG* gene, is a regulatory protein essential for activation of the ubiquitous transcription factor NF-κB [81,82]. Children with NEMO-related defects present variable levels of impaired host defenses, with severe susceptibility to IPD [83,84,85,86]. Patients with these disorders mount a weak inflammatory response with delayed fever or minimal change in inflammatory markers (e.g., leukocytosis and C reactive protein levels in serum), which may explain the mild inflammatory response elicited in vivo in these patients [87]. It is worth noting that patients with NEMO defects have persistent absence of anti-pneumococcal polysaccharides antibodies after naturally occurring pneumococcal infections and after challenge with polyvalent pneumococcal polysaccharide vaccine, whereas some IRAK-4-deficient patients do [82,87,88].

#### 5.1.2. Acquired Factors of Susceptibility to IPD

*S. pneumoniae* is overwhelmingly the most common infecting organism in functional or anatomic asplenic patients, accounting for 50–90% of isolates from blood cultures in many cohorts of patients, particularly in younger patients with sickle cell anemia [89]. Mortality from IPD in asplenic patients is more than 50% [90]. As the major site for T-cell independent antibody responses to bacteria and splenic mononuclear phagocytes, the spleen plays a critical role in controlling pneumococcal infection. Patients with asplenia have reduced levels of IgM memory B cells and IgM anti-pneumococcal antibodies, causing reduced ability to produce protective antibodies against polysaccharide antigens, and hence, possible vaccine failure [91,92].

Several studies have shown that HIV-infected individuals and adults have a significantly higher risk of acquiring *S. pneumoniae* and developing recurrent IPD [93,94]. Although active antiretroviral therapy significantly reduces the overall burden of IPD in HIV-positive populations, the risk of IPD remains 35 times higher in HIV-infected individuals than in non-HIV-infected adults [95]. Several studies have underlined the increased susceptibility to IPD in respiratory viruses infected patients, including influenza and respiratory syncytial viruses, especially in children [96,97,98]. Moreover, patients being treated for underlying solid or hematologic malignancies have high rates of invasive pneumococcal disease, although, interestingly, less than one-fifth of these infections occur during periods of neutropenia [99,100]. 

### 5.2. Impact on Anti-Pneumococcal Vaccination Strategies

Systematic immunological exploration in patients hospitalized for recurrent IPD is advocated. Levels of plasma Ig and IgG subclasses should be determined, especially in children who have a history of recurrent infections. In addition, screening of component complement deficiencies can be accomplished by an assessment of total complement function (CH50). Splenic function should be evaluated. In case of inherited immune deficiencies, siblings should also be examined. When detected, prophylactic measures are required to prevent infection. Based on the type of abnormality detected, these prophylactic measures fall into the following major axes:

- Vaccination. Vaccination against pneumococcal disease is safe and strongly recommended. Patients should receive sequential pneumococcal vaccination. Two types of vaccine against invasive pneumococcal disease are available, the pneumo-13V-conjugate vaccine (PCV-13) and the pneumo-polysaccharide-23V (PPV-23). Because these distinct types of vaccine stimulate immune responses somewhat differently, the criteria for protection from invasive pneumococcal disease are not the same for both. It is now recommended that initial vaccination with PCV-13 in children at high risk for severe pneumococcal infection should be followed by PPV-23 immunization starting at 24 months of age. This immunization should be given at least 8 weeks after the last PCV. A second dose of PPV-23 is recommended 5 years after. In patients older than 65 years, one dose of PCV-13 should be followed by PPV-23 at 6 to 12 months later. If PPV-23 was given first, PCV-13 is recommended to be given at least 12 months later. These approaches take advantage of the priming effect of PCV-13 and avoid the hypo-responsiveness to vaccination that might be caused by the PPV-23 [101]. However, hypo-responsiveness has been suggested to occur when plain polysaccharide vaccine is used regardless of the order of administration [102]. Household and other close contacts of persons with altered immunocompetence should also receive age-appropriate *S. pneumoniae* vaccines to minimize the risk of transmission to the immunocompromised contact [103,104]. *S. pneumoniae* has more than 90 serotypes. Although immunization may induce cross-protection against serotypes responsible for the majority of invasive infections, the vaccination fails to protect against other serotypes.
- Prophylactic antibiotics. Penicillin V is the most frequently used antibiotic [105]. Nevertheless, there is no international consensus on when to discontinue prophylaxis [106]. Furthermore, poor adherence to taking daily medications, the global spread and the potential for selection of penicillin-resistant organisms remain unresolved problems [105,107].
- Immunoglobulin replacement therapy. In most forms of antibody deficiency, the mainstay of therapy can be categorized by immunoglobulin (Ig) replacement to provide a protective serum IgG level [108]. Therapeutic IgG, which is usually needed for the duration of the patient’s life, are administered by intravenous (400 to 600 mg/kg every 3 to 4 weeks) or subcutaneous (100 to 150 mg/kg per week) routes to regularly ensure IgG trough levels in the normal range [109].
- Patient education. It is of utmost importance that individuals with altered immune competence be informed and educated about their increased risk for serious, life-threatening infections and understand the importance of seeking prompt medical attention should situations of risk arise (e.g., high fever). When traveling, especially to high-risk geographic areas, a prior consultation is necessary to receive recommendations and update vaccinations.

## 6. Susceptibility to Invasive GBS Infections

Group B *streptococcus* (GBS) is a leading cause of neonatal and infant sepsis and meningitis globally [110,111]. GBS can also cause stillbirths, prematurity and disease in pregnant women, immunocompromised adults and the elderly, but the highest incidence of disease is in neonates and young infants [112]. 

### 6.1. Genetic and Acquired Susceptibilities to Invasive GBS Disease

The susceptibility of neonates to GBS is correlated with a deficiency of maternal (transplacental)-specific antibody and the intrinsically immature immune system of neonates [113]. Moreover, GBS infections in nonpregnant adults typically present when the host is in an immunocompromised or relatively compromised state, such as diabetes, cancer, HIV, with diabetes being the predominating underlying condition [114,115,116]. The search for monogenetic immunodeficiency disorders underlying susceptibility to invasive GBS infections has only been partially successful so far. One patient with very late-onset GBS sepsis suffering from IRAK-4 deficiency has been reported, supporting that cellular innate immunity and the TLR system are important for resistance against GBS [117].

The severity of disease can be attributed, at least in part, to the virulence of the strain and its ability to avoid immunological clearance and adapt to changing environments throughout disease progression. Indeed, the ST-17 lineage responsible for severe neonatal disease, has a number of ST-17-specific genes that may contribute to its ability to cause meningitis [118]. 

### 6.2. Impact on Preventive Strategies

Intrapartum antibiotic prophylaxis (IAP) is the only preventive strategy currently available for the prevention of perinatal GBS early-onset disease (occurring from day 0 to day 6 of life) [117,119,120].

However, IAP coverage has no impact on late onset disease (LOD, which occurs from day 7 to 90 of life), stillbirths and prematurity due to GBS, as well as a limited impact on disease in pregnant women and it might be an issue for antimicrobial resistance [121,122]. Implementing a suitable vaccine for pregnant women could provide effective protection to those forms of invasive disease that cannot be prevented with IAP or where IAP is not feasible. This preventive strategy has been identified as a priority by WHO. Based on specific capsular polysaccharide antigens, 10 serotypes of GBS have been described. A hexavalent GBS glycoconjugate vaccine that covers the major six serotypes responsible for 99% of GBS infections is the most advanced vaccine candidate. Preclinical and human phase I and II studies have been completed, revealing the safety and immunogenicity of these vaccines [123,124,125]. However, a large number of participants would be required to undertake Phase III clinical efficacy trials. Protein vaccines that might confer protection irrespective of serotype, are in earlier stages of development. Future use of these vaccines raises the question of the adherence of pregnant women to routine vaccination. 

## 7. Conclusions

Several inherited or acquired risk factors are responsible for increased susceptibility to invasive bacterial diseases (Table 1). The investigation of patients with repeated invasive bacterial diseases and patients who developed these infections with unusual isolates is recommended. The genetic dissection of inherited factors will shed light on the molecular and cellular mechanisms underlying protective immunity to bacterial pathogens, and will improve our knowledge on the interaction of the pathogen with the human immune system to pave the way for the development of new, more appropriate treatments. Furthermore, early diagnosis and proper management of immune deficiencies are essential to avoid permanent damage and serious infectious complications. In addition to vaccination, antibiotic chemoprophylaxis (including intrapartum antibiotic prophylaxis for GBS infections) should be strongly considered. However, prolonged chemoprophylaxis using broad-spectrum antibiotics may select resistant bacterial isolates, increasing the risk of selective colonization with resistant isolates. Avoiding, when possible, the use of large-spectrum antibiotics and using vaccines, when available, can contribute to reducing antimicrobial resistance by reducing the selective pressure and preventing transmission of resistant isolates. Safe vaccination, when available, should be encouraged among high-risk patients and their close contacts to prevent these infectious diseases.

## Figures and Tables

**Table 1:** Congenital and acquired deficiencies and anatomic conditions that may predispose to meningococcal, pneumococcal, *H. influenzae* or GBS invasive infections requiring prevention strategies against invasive bacterial diseases (adapted from references cited in the text).

Involved Deficiency	Transmission (if Known/Applicable)	Estimated Frequency	Clinical Aspects (if Known)	Risk for Invasive Bacterial Infections
Early complement components (C1–C4)	Mendelian recessive but dominant for C1q inhibitor deficiency	C1q and C2 (1:20,000 to 100,000). Rare for the other components	Hereditary angioedema (C1q), systemic lupus erythematosus, glomerulonephritis	Yes, IMD, IPD, IHiD, GBS
Late complement components (C5–C9)	Mendelian recessive. Acquired with anti C-5 treatment	Variable ethnically C6 in Africans and Afro-Americans (1:20 000). C9 in Japanese (1:1000)		Yes, in particular, repeated IMD
Mannose-binding lectin	Non- Mendelian	5% in Caucasian subjects		Debated
Complement regulators (Properdin, Factors B, D, I and H)	Mendelian recessive (X-linked for properdin)	Rare	Atypical hemolytic uremic syndrome, paroxysmal nocturnal hemoglobinuria (PNH), age-related macular degeneration (AMD)	Yes
Antibody (B cell) immunodeficiencies	Heterogeneous		Primary and secondary impairment of antibody production	Yes, in particular IPD, IHiD
Asplenia			Functional or anatomical	Yes. Repeated infections with capsulated bacteria
Toll-like receptor signaling (IRAK-4, MyD88, NEMO)			Innate immunity signaling and Immunodeficiency	Yes, in particular IPD, GBS
Other polymorphisms (IL-10 promoter, Fc-gamma RIIa receptor, TIRAP)			Innate immunity signaling and Immunodeficiency	Yes, in particular IHiD

IPD Invasive Pneumococcal Disease. IHiD Invasive *Haemophilus influenzae* Disease. IMD Invasive Meningococcal Disease. GBS Group B Streptococci. IRAK-4 Interleukin-1 receptor-associated kinase 4. MyD88 Myeloid differentiation primary response 88. NEMO Nuclear factor-kappa B Essential Modulator. TIRAP Toll-interleukin 1 receptor (TIR) domain containing adaptor protein.

## Data Availability

Not applicable.
